# Gene dysregulation in peripheral blood of moyamoya disease and comparison with other vascular disorders

**DOI:** 10.1371/journal.pone.0221811

**Published:** 2019-09-18

**Authors:** Xing Peng, Zhengshan Zhang, Dongqing Ye, Peiqi Xing, Zhengxing Zou, Hongxing Lei, Lian Duan

**Affiliations:** 1 CAS Key Laboratory of Genome Sciences and Information, Beijing Institute of Genomics, Chinese Academy of Sciences, Beijing, China; 2 University of Chinese Academy of Sciences, Beijing, China; 3 Department of Neurosurgery, the Fifth Medical Center, Chinese PLA General Hospital, Beijing, China; 4 Center of Alzheimer’s Disease, Beijing Institute for Brain Disorders, Beijing, China; The Second Affilated Hospital, Zhejiang University School of Medicine, CHINA

## Abstract

**Objective:**

Moyamoya disease (MMD) is a chronic occlusive cerebrovascular disease with unknown etiology, sharing many similar clinical symptoms with other vascular disorders. This study aimed to investigate gene dysregulation in peripheral blood of MMD and compare it with other vascular disorders.

**Methods:**

Transcriptomic profiles of 12 MMD patients and 8 healthy controls were obtained using RNA sequencing. Differentially expressed genes (DEGs) were identified and several were validated by quantitative real-time PCR in independent samples. Biological pathway enrichment analysis of DEGs and deconvolution of leukocyte subsets in peripheral blood were performed. Expression profiles for other vascular diseases were downloaded from public database and consistent DEGs were calculated. Gene set enrichment analysis (GSEA) was conducted to compare gene dysregulation pattern between MMD and other vascular diseases.

**Results:**

A total of 533 DEGs were identified for MMD. Up-regulated genes were mainly involved in extracellular matrix (ECM) organization, whereas down-regulated genes were primarily associated with inflammatory and immune responses. As for cell populations, significantly increased naïve B cells and naïve CD4 cells as well as obviously decreased resting natural killer cells were observed in peripheral blood of MMD patients. GSEA analysis indicated that only up-regulated genes of ischemic stroke and down-regulated genes of coronary artery disease and myocardial infarction were enriched in up-regulated and down-regulated genes of MMD, respectively.

**Conclusion:**

Dysregulated genes in peripheral blood of MMD mainly played key roles in ECM organization, inflammatory and immune responses. This gene dysregulation pattern was specific compared with other vascular diseases. Besides, naïve B cells, naïve CD4 cells and resting natural killer cells were aberrantly disrupted in peripheral blood of MMD patients. These results will help elucidate the complicated pathogenic mechanism of MMD.

## Introduction

Moyamoya disease (MMD) is a chronic intracranial vascular disease characterized by progressive narrowing or occlusion at the terminal part of the internal carotid artery (ICA) and its nearby branches. The disease is also accompanied by formation of an abnormal vascular network at the base of the brain, which looks like ‘a puff of smoke’ on angiography[1]. Ischemic attack and intracranial hemorrhage are the two main clinical manifestations, the former of which predominantly happens in pediatric MMD, whereas both in adult MMD[2–5].

Despite much progress made over the past few decades, the etiology and pathogenesis of MMD remain largely unknown. Multiple factors involving genetic, immunological, and angiogenic aspects have been reported to be associated with MMD pathology. *RNF213* in the 17q25-ter region, which encodes a ring finger protein with both E3 ubiquitin ligase activity and ATPase activity, has been confirmed to be a high susceptibility gene for MMD in East Asian populations[6–11]. Although knockdown of *RNF213* in zebrafish induced formation of aberrant vessels in the head region[7], *RNF213*-deficient mice did not subsequently develop MMD as expected[12]. Besides genetic aspect, environmental factors could also play a key role in the pathogenesis of MMD. On one hand, an increasing number of studies have revealed a significant association between MMD and immunological diseases, implying a potential immune component in the pathogenesis of MMD[13–16]. On the other hand, many recent studies have also focused on abnormal revascularization in MMD patients, which is probably a protective response to the ischemic and hypoxia cerebral environment[17]. For instance, elevated expression of angiogenic factors such as basic fibroblast growth factor (bFGF), vascular endothelial growth factor (VEGF), and transforming growth factor-β1 (TGF-β1) in cerebrospinal fluid, plasma or serum was observed in MMD patients[18–21].

MMD, though as an uncommon cerebrovascular disorder, shares many similar clinical manifestations with other vascular diseases due to narrowing or blockage of different arteries. Previous findings from histopathological and genetic studies revealed that MMD was associated with atherosclerotic disease[22, 23]. Cases of coronary heart disease in patients with MMD were also widely reported[24–29]. Also, a recent retrospective study revealed that 4.6% of the MMD patients were found to have coronary heart disease, suggesting a possible internal association between MMD and coronary heart disease[27]. Therefore, MMD seems to be a systemic vasculopathy involving not only intracranial arteries but also extracranial arteries[30]. Gene expression profiling of peripheral blood has been widely utilized to investigate a variety of vascular diseases, facilitating the discovery of effective disease biomarkers for earlier diagnosis and accurate classification of disease subtypes[31–36]. Thus, comparing gene expression pattern in peripheral blood between MMD and other vascular disorders could be an effective approach to investigate the potential association among them.

In the present study, we for the first time applied high-throughput RNA sequencing (RNA-Seq) to investigate gene dysregulation in peripheral whole blood of patients with MMD and compared it with other vascular disorders to identify MMD-specific signature on the transcriptional level. In addition, biological pathway enrichment analysis of dysregulated genes and deconvolution of leucocyte subtypes in peripheral whole blood of patients with MMD were performed. Our study may help clarify the obscure pathogenic mechanism of MMD and elucidate the complicated association between MMD and other vascular disorders. It is also useful for screening for effective disease biomarkers.

## Materials and methods

### Blood sample collection

Participants in this study were recruited from the Department of Neurosurgery at the Fifth Medical Center of Chinese PLA General Hospital from March 2017 through July 2017. Detailed clinical data of all the participants were collected. Besides history of present illness, past history and family history of patients were also enquired. Subsequently, cerebral magnetic resonance imaging (MRI), magnetic resonance angiography (MRA), cerebral magnetic resonance perfusion imaging and digital subtraction angiography (DSA) were performed for all the patients and Suzuki stage of MMD was confirmed[37]. At the same time, blood lipids, antinuclear antibody spectrum, thyroid function, thyroid autoantibodies, anti-O and rheumatoid factors were examined to help make a differential diagnosis of definitive MMD. Based on all these data, patients with underlying diseases such as atherosclerosis, autoimmune disease, brain tumors, Down’s syndrome, meningitis, neurofibromatosis type 1, and sickle cell disease, or those having a history of head injury or suffering stroke (ischemic or hemorrhagic stroke) within one month were excluded. Finally, a definite diagnosis was made according to the Guidelines for Diagnosis and Treatment of Moyamoya Disease (Spontaneous Occlusion of the Circle of Willis)[37]. In addition, unilateral and familial MMD patients were also excluded from this study. Healthy controls without history of diseases such as vascular diseases, immune system diseases, hypertension, hyperlipidemia, and diabetes mellitus were also recruited. All subjects were Han Chinese. 2.5 ml peripheral whole blood was drawn into the PAXgene Blood RNA Tubes (Qiagen) from each subject and stored in -80°C until use. Written informed consent were obtained from all participants. This study was performed in accordance with the ethical guidelines of the 1975 Declaration of Helsinki and was approved by the ethics committee of the Fifth Medical Center of Chinese PLA General Hospital.

### Public blood transcriptome datasets

Gene expression profiling datasets for vascular disorders including ischemic stroke (IS), atherosclerosis (ATS), familial hypercholesterolemia (fHC), coronary artery disease (CAD) and myocardial infarction (MI) were searched and downloaded from NCBI Gene Expression Omnibus (GEO, https://www.ncbi.nlm.nih.gov/geo/) database. Of note, we grouped MI as an independent type because MI is the most serious type of CAD. These vascular disorders are selected because they all present with or will finally lead to progressive narrowing or occlusion of involved arteries, sharing many similar clinical symptoms with MMD. In addition, extensive studies have suggested an association between MMD and these vascular disorders. Specially, we included fHC in our analysis because longstanding fHC could lead to massive atheromatous plaques and finally result to the stenosis or occlusion of the involved arteries. Key words including ‘ischemic stroke’, ‘atherosclerosis’, ‘hypercholesterolemia’, ‘coronary artery disease’ and ‘myocardial infarction’ were used for preliminary search in GEO database, respectively. Only datasets including both patients and healthy controls were retained. All datasets were gene expression profiling in peripheral whole blood by microarray. Details of the above datasets are summarized in **S1 Table**.

### RNA extraction and RNA-Seq

Total RNA in peripheral whole blood from MMD patients and healthy individuals was extracted using PAXgene Blood RNA Kit (Qiagen) according to the manufacturer’s instructions. RNA concentration and purity were measured by NanoDrop 8000 (Thermo Scientific). RNA integrity was determined by agarose gel electrophoresis and Agilent Bioanalyzer 2100 (Agilent). Stranded RNA-Seq libraries were prepared using KAPA RNA HyperPrep Kit with RiboErase (KAPA Biosystems) and subjected to paired-end (2*150bp) sequencing on Illumina HiSeq 4000. Raw data were deposited in the Genome Sequence Archive (https://bigd.big.ac.cn/gsa-human)[38] in BIG Data Center, Beijing Institute of Genomics, Chinese Academy of Sciences, under accession number HRA000065.

### Quantitative real-time PCR

First strand cDNA was synthesized from total RNA using RevertAid First Strand cDNA Synthesis Kit (Thermo Scientific) with oligo(dT)_18_ primer. Quantitative real-time PCR (qRT-PCR) was performed using PowerUp SYBR Green Master Mix (Thermo Scientific) on Bio-Rad CFX96 PCR instrument. PCR reactions were performed in 10 μl volume under the following conditions: 50°C for 2 min, 95°C for 2 min, followed by 40 or 45 cycles of 15 s at 95°C and 1 min at 60°C. *B2M* was used as the internal control. Relative gene expression was calculated by the 2^-ΔΔCt^ method. Primer sequences are listed in **S2 Table**. Graphs for qRT-PCR results were generated using GraphPad Prism 6.01 (GraphPad Software, Inc.).

### Bioinformatics analysis

RNA-Seq raw reads were preprocessed with cutadapt[39]. Quality control was performed using FastQC (http://www.bioinformatics.babraham.ac.uk/projects/fastqc/). STAR[40] was used for reads mapping. Raw reads count was calculated using HTSeq[41]. Differentially expressed genes (DEGs) were identified by DESeq2[42] with *p* < 0.05 and fold change ≥ 1.5 and sex factor was incorporated in the generalized linear model considering that many sex-biased genes existed in the human peripheral blood transcriptome[43].

For Affymetrix microarray, CEL files were downloaded and subjected to background correction and normalization using R package RMA[44]. For other microarray platforms, expression matrix was downloaded and quantile normalization was performed. Probes with intensity value below 50 in more than half of the samples were removed. Expression values were log2 transformed. DEGs were determined using R package RankProd[45] with *p* < 0.05 and fold change ≥ 1.5. DEGs for each vascular disorder were figured out separately based on occurrence count of the gene in the datasets.

Gene Ontology (GO) and Kyoto Encyclopedia of Genes and Genomes (KEGG) pathway enrichment analysis were performed using online tool DAVID (Database for Annotation, Visualization and Integrated Discovery, https://david.ncifcrf.gov/). Co-expression network of the top 100 DEGs (ranked by *p*-values) in peripheral blood of MMD was constructed using GEO dataset GSE48348[46]. Only edges with absolute Pearson correlation coefficient > 0.7 in the network were retained. Cell-type Identification By Estimating Relative Subsets Of RNA Transcripts (CIBERSORT)[47] was used for the deconvolution of cell types in peripheral whole blood. Gene set enrichment analysis (GSEA, version 3.0, Broad Institute, Inc.) was used to compare gene dysregulation pattern between MMD and other vascular disorders. Ten gene sets including corresponding up-regulated and down-regulated genes of 5 vascular disorders served as gene sets used in GSEA analysis. Gene sets with less than 15 genes were removed. Similarities on gene dysregulation pattern between MMD and other vascular diseases were evaluated by the significance of enrichment. Graphs were generated using R statistical software 3.3.2 (R core team) and Cytoscape 3.3.0 (Cytoscape Consortium).

### Statistical analysis

Data are represented as the mean ± SEM from three independent experiments unless otherwise specified. Unpaired student’s *t*-test was used for statistical evaluation. *p* < 0.05 was considered statistically significant. Statistical analysis was performed using R statistical software 3.3.2 (R core team).

## Results

### Clinical characteristics of MMD patients and healthy controls

A total of 66 participants (MMD patients, n = 35; healthy controls, n = 31) were retained in this study. All patients were definitely diagnosed with bilateral sporadic MMD based on diagnostic imaging results and other clinical information. Initial symptoms of MMD patients included transient ischemic attack (TIA), cerebral infarction (CI), intraventricular hemorrhage (IVH), and intracerebral hemorrhage (ICH). Of all the 35 MMD patients, over half (62.9%) presented with TIA symptom. Subjects were subsequently divided into two groups, one of which (MMD patients, n = 12; healthy controls, n = 8) was used for gene expression profiling with RNA-Seq and the other group (MMD patients, n = 23; healthy controls, n = 23) was used for validation with qRT-PCR. Considering for the limited sample size, we made certain basic inclusion criteria for samples in the RNA-Seq group. These criteria included: (1) the male to female ratio of MMD patients should be 1:1, (2) at least 50% of patients presented with TIA symptom, (3) patients with other three initial symptoms should have equal number, (4) all subjects should be free of other diseases such as hypertension, hyperlipidemia, and diabetes mellitus. This criteria not only reflected the global distribution of initial symptoms in MMD patients to some extent but also avoided uncertain influences on the final result from other diseases. There was no significant difference with respect to age between MMD patients and healthy subjects (45.75 ± 9.24 vs 37.17 ± 12.84, *p* = 0.12) in the RNA-Seq group. Mean age tended to be older in MMD patients than in healthy controls (34.61 ± 14.81 vs 44.35 ± 8.42, *p* = 0.01) from the qRT-PCR group. However, our previous study has shown that effect of age on gene expression in peripheral blood was so weak that expression of only six genes including *NELL2*, *CCR2*, *CCR7*, *MYC*, *LTB* and *FAM102A* displayed weak negative correlation (Pearson correlation coefficients varying from -0.29 to -0.47) with age in four public datasets[48]. Baseline characteristics of all participants were shown in **Table 1**. Suzuki stage and past history of corresponding subjects were also shown. Data of other clinical tests on MMD patients were shown in **S3 Table**.

**Table 1:** Clinical characteristics of MMD patients and healthy controls.

Group	RNA-Seq		qRT-PCR	
Items	MMD (n = 12)	Healthy control (n = 8)	MMD (n = 23)	Healthy control (n = 23)
Sex				
Male	6	3	8	15
Female	6	5	15	8
Age (mean ± sd)	37.17 ± 12.84	45.75 ± 9.24	34.61 ± 14.81	44.35 ± 8.42
Initial symptom				
TIA	6	NA	16	NA
CI	2	NA	6	NA
IVH	2	NA	0	NA
ICH	2	NA	1	NA
Family history of MMD	0	NA	0	NA
Suzuki stage (left—right)				
I	0–0	NA	3–1	NA
II	0–0	NA	3–8	NA
III	6–5	NA	7–3	NA
IV	2–4	NA	5–6	NA
V	3–3	NA	5–2	NA
VI	1–0	NA	0–3	NA
Bilateral lesion	12	NA	23	NA
Past history				
hypertension	0	0	3	0
hyperlipidemia	0	0	1	0
diabetes mellitus	0	0	2	0

TIA = transient ischemic attack; CI = cerebral infarction; IVH = intraventricular hemorrhage; ICH = intracerebral hemorrhage.

### Gene dysregulation in peripheral blood of MMD patients

**Fig 1:**
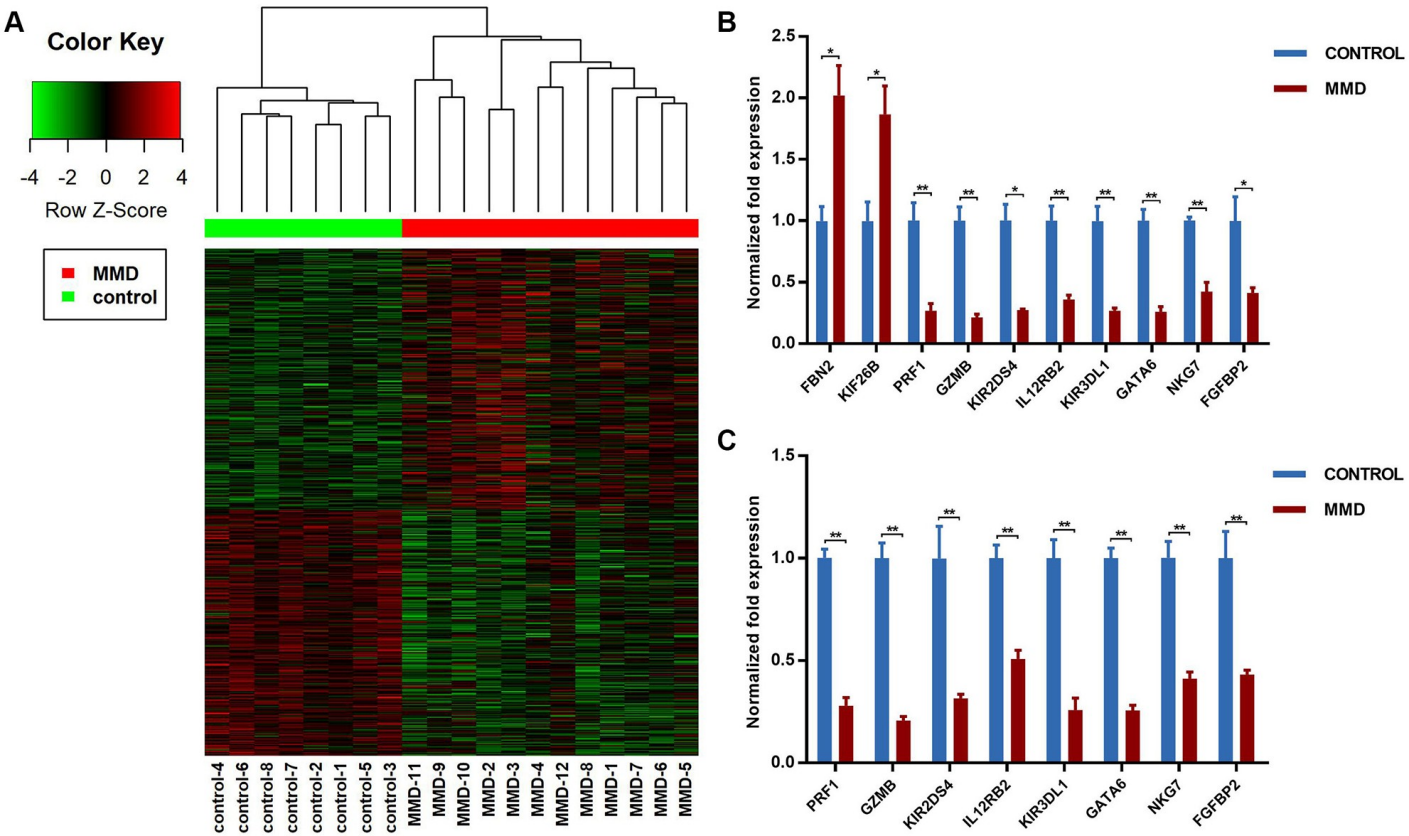
Dysregulated genes in peripheral blood of MMD. **(A)** Heat map and hierarchical clustering demonstrating the expression pattern of DEGs between MMD patients and healthy controls. The color key at the top left indicates relative gene expression. Red and green colors represent higher and lower gene expression levels, respectively. **(B-C)** Validation of RNA-Seq results using qRT-PCR in RNA-Seq samples including 12 MMD patients and 8 heathy controls and in independent samples including 23 MMD patients and 23 healthy controls. Normalized fold expression of mRNA are shown. Expression values are first normalized to *B2M* (internal control) and then plotted relative to corresponding healthy controls that are set as 1 for each gene. Error bar represents mean ± SEM (n = 3 independent experiments). Unpaired student’s *t*-test is used to calculate significant differences (*p < 0.05, **p < 0.01).

Gene expression profiling in peripheral blood of MMD patients and healthy controls was performed using RNA-Seq. As a result, 533 DEGs including 275 up-regulated and 258 down-regulated were identified. Among them, 394 DEGs were protein-coding genes and 139 DEGs were non-coding genes. Hierarchical cluster analysis based on gene expression of the 533 DEGs demonstrated that MMD patients and healthy controls were clearly separated into two clusters (**Fig 1A**). Furthermore, we ranked the 533 DEGs according to their *p*-values and found that many of the top 100 DEGs were immune or inflammation-related genes. There were also many genes associated with extracellular matrix (ECM) organization in the top 100 DEGs. Subsequently, we further selected 10 genes (*FBN2*, *KIF26B*, *PRF1*, *GZMB*, *KIR2DS4*, *IL12RB2*, *KIR3DL1*, *GATA6*, *NKG7*, *FGFBP2*) based on their fold change values and associations with immune response and ECM organization from the top 100 DEGs and confirmed their differential expression in RNA-Seq samples by qRT-PCR (**Fig 1B**). Finally, we also validated differential expression of all the above 10 DEGs except for *FBN2* and *KIF26B* in the independent qRT-PCR group (**Fig 1C**). To exclude potential influences on our validation result from sex factors and other clinical factors, we removed those subjects with hypertension, hyperlipidemia, and diabetes mellitus, and then performed differential expression analysis with male and female subjects, respectively. As a result, those genes we selected were also significantly dysregulated in both the male and the female cohorts (**S1 Fig**). Furthermore, correlation between gene expression of these ten genes and sex factor were very weak (absolute Spearman correlation coefficient < 0.5). Therefore, these genes were indeed aberrantly expressed. These results suggested that MMD, an uncommon cerebrovascular disorder, could display significant gene dysregulation in peripheral blood.

### Gene Ontology and KEGG pathway enrichment analysis

**Fig 2:**
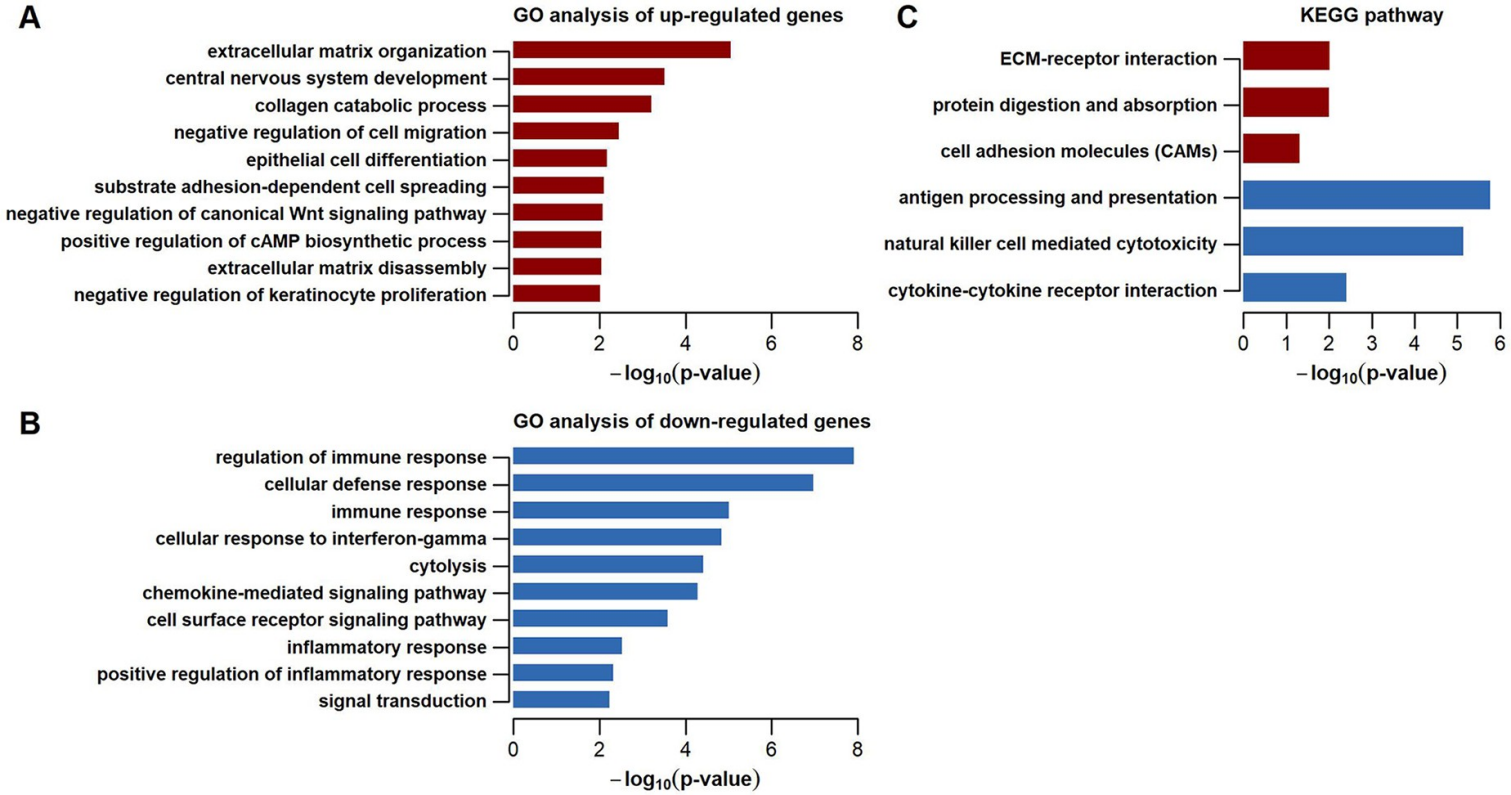
GO and KEGG pathway enrichment analysis of dysregulated genes in peripheral blood of MMD patients. Top 10 GO biological processes enriched in up-regulated and down-regulated genes are shown in **(A)** and **(B)**, respectively. Top 6 KEGG pathways over-represented in dysregulated genes (dull-red: up-regulated; blue: down-regulated) are shown in **(C)**. GO = Gene Ontology; KEGG = Kyoto Encyclopedia of Genes and Genomes; ECM = extracellular matrix.

To better understand biological processes and pathways that the dysregulated genes may be involved in, we performed GO and KEGG pathway enrichment analysis. GO analysis suggested that up-regulated genes were mainly involved in biological processes including ECM organization, central nervous development, collagen catabolic process, negative regulation of cell migration, and epithelial cell differentiation. (**Fig 2A**), whereas down-regulated genes primarily played a role in immune and inflammatory responses, cellular defense response, cellular response to interferon-gamma, cytolysis, and chemokine-mediated signaling pathway (**Fig 2B**). Likewise, KEGG pathway enrichment analysis showed that ECM-receptor interaction, protein digestion and absorption, and cell adhesion molecules were pathways over-represented in up-regulated genes, while antigen processing and presentation, natural killer cell mediated cytotoxicity, and cytokine-cytokine receptor interaction were enriched pathways in down-regulated genes (**Fig 2C**). Thus, these enriched biological processes or pathways mainly involving ECM organization, immune and inflammatory responses, may be tightly associated with the complicated pathogenic process of MMD. Furthermore, top DEGs in these enriched pathways may provide potential value for earlier diagnosis of MMD and monitoring of disease process.

### Gene co-expression network

**Fig 3:**
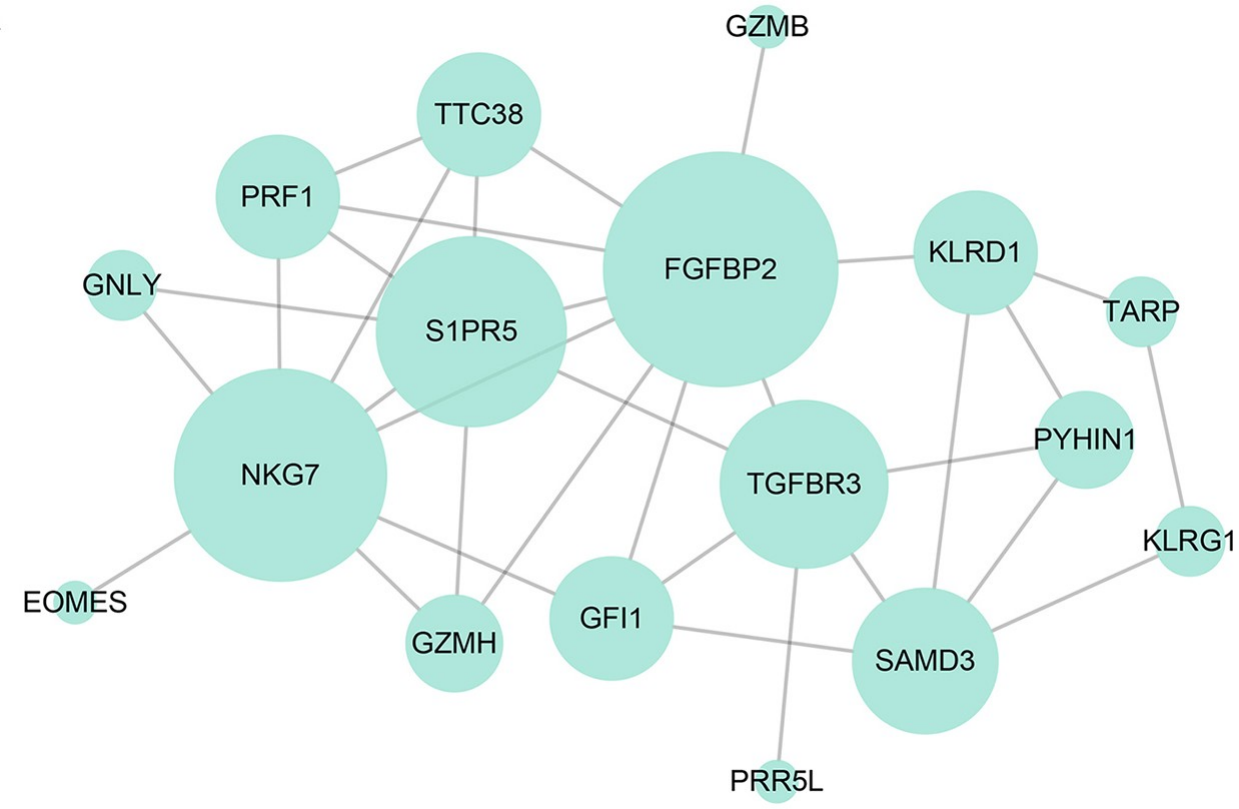
Gene co-expression network of the top 100 DEGs between MMD patients and healthy controls. Co-expression network was constructed using expression profiles of 734 healthy subjects from GEO dataset GSE48348. Only edges with Pearson correlation coefficient over 0.7 are retained and ring size indicates number of edges. GEO = gene expression omnibus.

To explore the internal relationships of dysregulated genes, co-expression network of the top 100 DEGs in MMD was constructed using expression profiles of 734 healthy subjects from GEO dataset GSE48348, considering the limited sample size in our RNA-Seq group. After removing edges with absolute Pearson correlation coefficient ≤ 0.7, several highly correlated hub genes such as *NKG7*, *S1PR5*, *FGFBP2*, and *TGFBR3* were retained in the core network (**Fig 3**). In addition, many genes in the network were immune and inflammation-related genes, suggesting a crucial role of immunological factors in the molecular mechanism behind MMD. Furthermore, dysregulation of several hub genes in the co-expression network (*NKG7*, *FGFBP2*, *PRF1*) were validated by qRT-PCR in our independent samples (**Fig 1C**).

### Disturbance of leukocyte populations in peripheral blood of MMD patients

**Fig 4:**
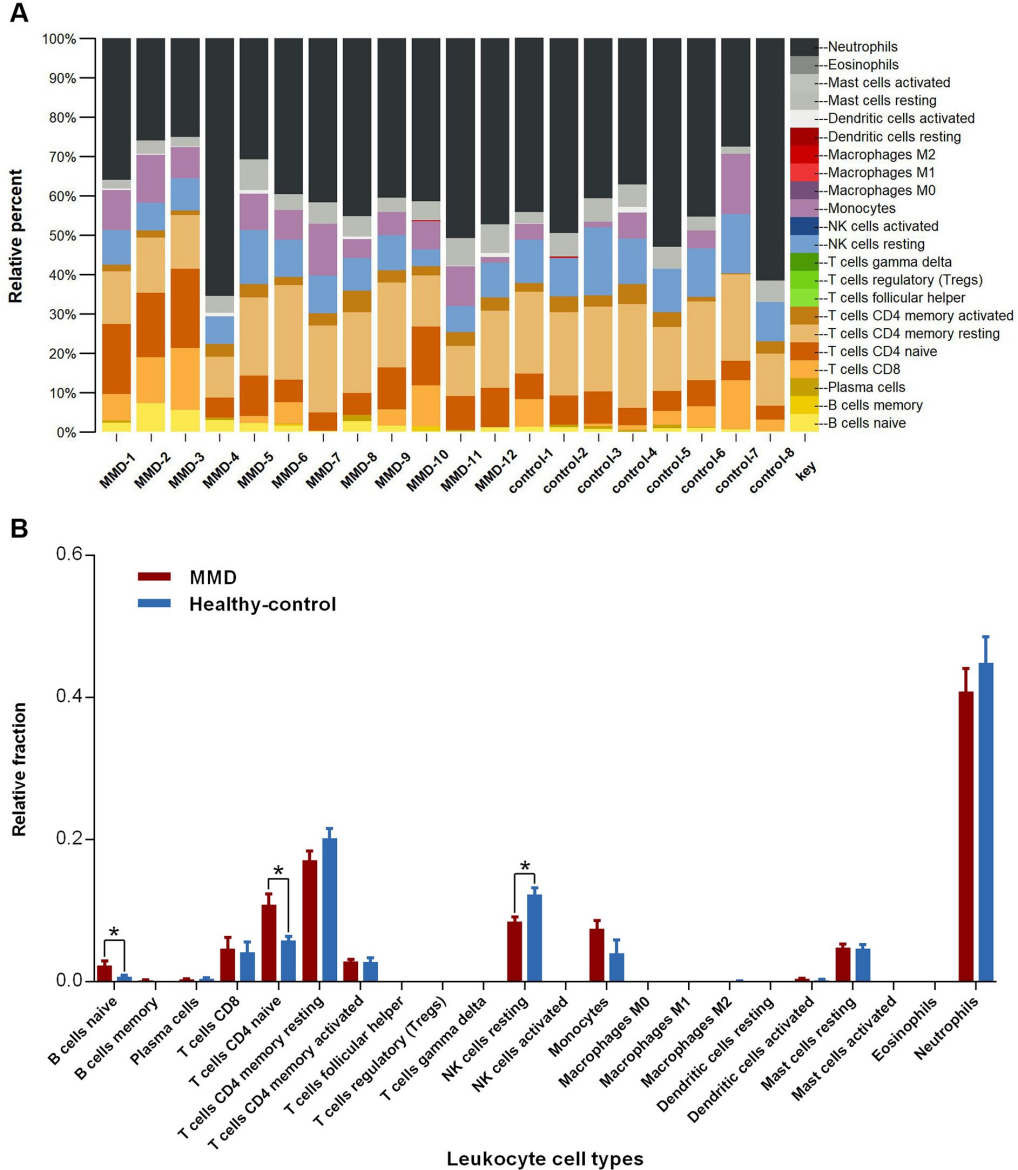
Deconvolution of leukocyte subtypes in peripheral blood of MMD patients and healthy controls. **(A)** Stacked bar chart demonstrates the relative percent of 22 leukocyte subtypes in each sample. **(B)** Relative fraction changes of 22 leukocyte subtypes between MMD patients and healthy controls. Unpaired student’s *t*-test is used to calculate significant differences (*p < 0.05).

Leukocytes are the most important cell types in peripheral blood for their involvement in the immune system. Since previous findings and findings in the present study supported an immune component in MMD pathogenesis, herein, we conducted cell type deconvolution to explore changes on proportion of leukocytes in peripheral blood of MMD patients. Proportion of 22 leukocyte subtypes in MMD patients and healthy controls from the RNA-Seq group was shown in **Fig 4A**. Neutrophil and resting memory CD4 T cell were the two most abundant cell types with a mean fraction of more than 50%. Unexpectedly, we found that most cell types did not show aberrant fluctuation on proportion in MMD patients when compared to healthy controls. However, proportion of naïve B cells and naïve CD4 cells significantly increased and that of resting natural killer cells significantly decreased in MMD patients (**Fig 4B**). Therefore, disturbance of these three cell types could be a potential effective indication to the MMD disease state.

### Comparison of gene dysregulation in peripheral blood of MMD with other vascular disorders

**Fig 5:**
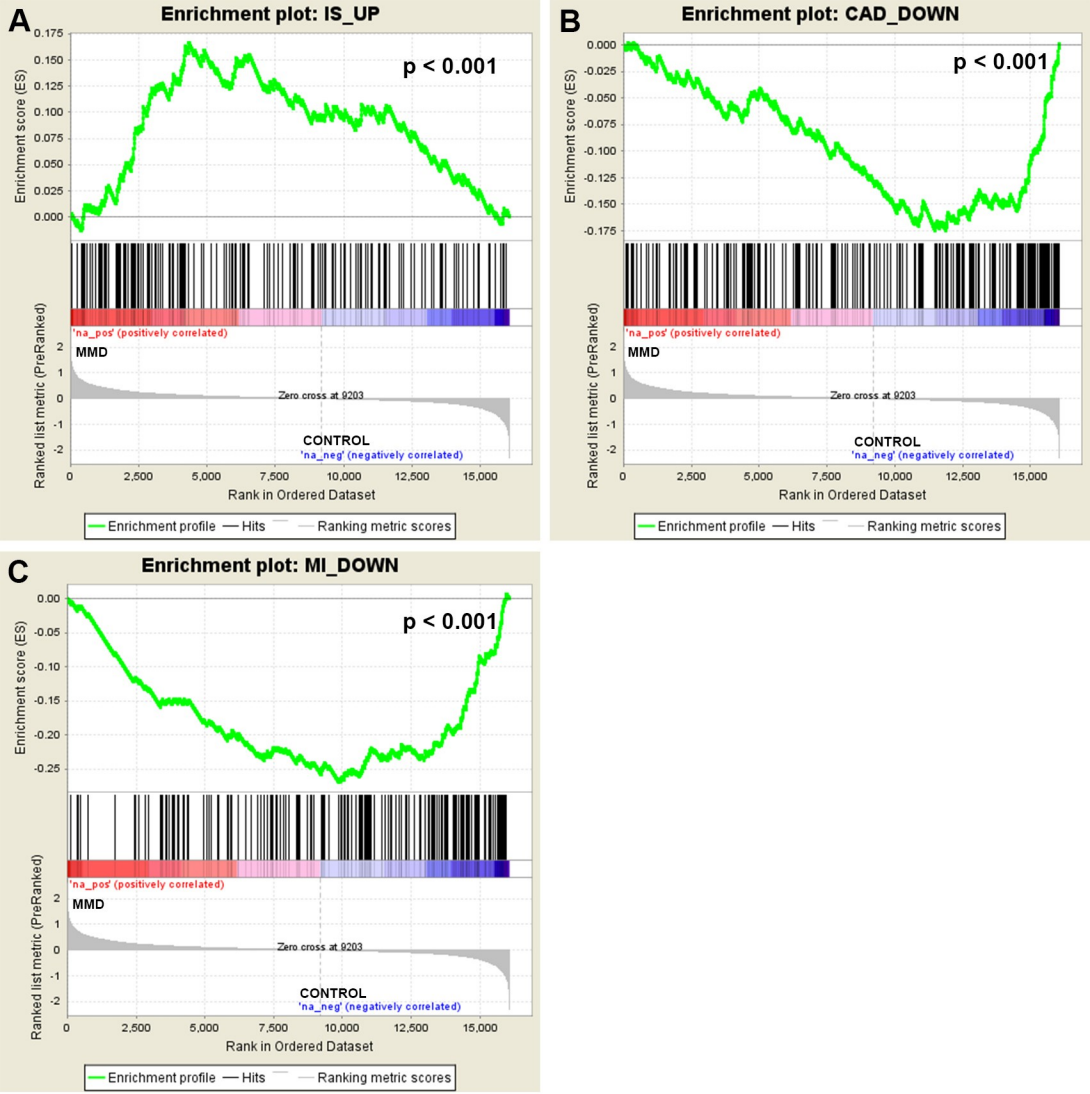
Comparing gene dysregulation in peripheral blood of MMD with other vascular disorders by GSEA. IS_UP (**A**), CAD_DOWN (**B**) and MI_DOWN (**C**) are the 3 most enriched gene sets. In each enrichment plot, genes are ranked according to log2 fold change value (i.e. from the most up-regulated to the most down-regulated genes between MMD patients and healthy controls). Vertical bars indicate the position of each gene in the gene set within the ranked list. Enrichment score (ES) is calculated by walking down through the ranked list. ES is increased when a gene is in the gene set and decreased when it is not. P value indicates the significance of ES for a gene set. GSEA = gene set enrichment analysis; IS_UP = up-regulated genes in ischemic stroke; CAD_DOWN = down-regulated genes in coronary artery disease; MI_DOWN = down-regulated genes in myocardial infarction.

Finally, we sought to compare gene dysregulation in peripheral blood of MMD with other vascular disorders. First, overlap of DEGs between each vascular disorder was calculated and we found that MMD shared only a few DEGs with other vascular disorders (**S4 Table**). Next, GO enrichment analysis with DEGs in each vascular disorder suggested that few enriched GO terms (biological processes) were shared by MMD and other vascular disorders. Cell migration (GO: 0016477) and immune response (GO: 0006955) were the top two overlapped GO terms between MMD and other vascular diseases (**S1 File**). Last, GSEA analysis was performed to further compare gene dysregulation pattern in peripheral blood of MMD with other vascular disorders. Ten gene sets that consisted of corresponding up-regulated and down-regulated genes of five vascular diseases were further reduced to 9 gene sets for GSEA analysis after gene set of down-regulated genes in atherosclerosis was removed due to small gene set size. We found that only genes up-regulated in ischemic stroke was significantly (*p* < 0.001) enriched in up-regulated genes of MMD, while only genes down-regulated in coronary artery disease and myocardial infarction were over-represented (*p* < 0.001) in down-regulated genes of MMD (**Fig 5**). Thus, some other vascular disorders could partially, however, not fully exhibit certain similar gene expression pattern in peripheral blood to that of MMD. Together, these results demonstrated that compared with other vascular disorders, MMD displayed a unique gene dysregulation pattern in peripheral blood, which was mainly associated with ECM organization, immune and inflammatory responses.

## Discussion

As a rare cerebrovascular disorder, MMD is characterized by progressive constriction or blockage at the terminal portion of ICA and main vascular branches in the circle of Willis[1]. Therefore, it is not practical to perform extensive studies by directly using the intracranial vessels involved in the pathogenesis of MMD. Peripheral blood circulating throughout the body not only can provide useful information about the lesion site of disease to some extent but also is easily accessible and thus can serve as a potential surrogate material for studying the molecular mechanism of MMD. In the present study, we performed RNA-Seq to profile gene expression in peripheral whole blood of patients with MMD and healthy controls. Aberrantly expressed genes were identified and several top DEGs involved in immune response and ECM organization were also validated in a cohort of independent samples with qRT-PCR. Matrix metalloproteinases (MMPs), a group of calcium-dependent zinc-containing endopeptidases, play critical roles in ECM remodeling, cell proliferation and migration, angiogenesis, and inflammatory processes[49]. Previous studies have examined protein expression of MMPs and their tissue inhibitors (TIMPs) in serum or plasma of MMD patients to investigate their potential roles in MMD pathogenesis[18, 50]. Here, we found MMD patients exhibited obviously higher expression of *MMP11* (*p* = 0.007), *MMP17* (*p* = 0.006), *MMP28* (*p* = 0.011) and *TIMP3* (*p* = 0.037) in peripheral blood than that of healthy controls. These results supported the hypothesis that disrupted balance between MMPs and TIMPs may be involved in the vascular stenosis or occlusion and the formation of abnormal vascular network in patients with MMD[18, 50].

MMD is a progressive occlusive cerebral vasculopathy with unknown etiology[1]. It is suspected that MMD could be a result secondary to inflammatory and immunological responses[13, 16, 51–54]. Kang et al. also reported that down-regulated genes in purified circulating smooth-muscle progenitor cells from peripheral blood in MMD patients were primarily related to immune response[55]. Consistent with this view, we demonstrated that up-regulated genes in peripheral blood of MMD patients were mainly involved in ECM organization, whereas down-regulated genes were significantly associated with immune and inflammatory responses (**Fig 2**). ECM, as a highly heterogeneous and dynamic composition, plays vital roles in many biological processes such as cell proliferation, cell migration, and cell adhesion. Moreover, ECM proteins and proteoglycans can interact with various growth factors like bFGF and VEGF to regulate many signal transduction pathways[56]. Thus, we suppose that disruption of ECM organization may somehow trigger the abnormal proliferation, differentiation and migration of vascular cells like smooth muscle cells in the blood vessel wall and subsequently result to the accumulation of fibrous substances, finally leading to the progressive vascular stenosis or occlusion in MMD. In addition, many of the hub genes such as *NKG7*, *FGFBP2*, *GZMH*, and *PRF1* in the gene co-expression network were associated with immune function (**Fig 3**). For instance, *PRF1* encodes a membrane pore protein, which allows the release of granzymes and subsequent cytolysis of target cells. These hub genes might act as drivers to disrupt regular immune pathway and eventually accelerate the progression of MMD. In addition, hub gene *TGFBR3* encodes the transforming growth factor (TGF) beta 3 receptor, which can act as a co-receptor with other members in the TGF beta receptor superfamily and cooperate with TGF-β1 to regulate the TGF signaling pathway. Furthermore, previous studies suggested that TGF-β1, an angiogenic factor and playing key roles in regulating expression of connective tissue genes[57–61], was associated with the pathogenesis of MMD[19]. This suggested that TGF-β1 and its receptors may function as key contributors to the fibrotic thickening of the intima, abnormal internal elastic lamina, and the excessive ECM deposits in the occlusive moyamoya vessels. Collectively, these results indicated ECM organization, inflammation and immunity played a vital role in the pathological process of moyamoya vasculopathy.

Changes on cell populations in MMD patients have been widely reported in the past. Thickening of the intimal resulting from excessive migration and proliferation of smooth muscle cells was one of the major histopathological findings of MMD[62]. Both increased and decreased level of circulating EPCs in patients with MMD were reported[63–66]. Weng et al. also revealed an increase of peripheral Treg and Th17 cells in MMD patients compared with healthy controls[67]. These findings suggested that multiple cell types may be involved in the pathogenesis of MMD. However, most of these studies applied fluorescence-activated cell sorting (FACS) technique to estimate abundance of only several cell types each time due to limited phenotypic markers. This limits exploration of proportion changes on circulating cell types on a large scale. Herein, we applied a computational method named CIBERSORT, which was based on gene expression profiles, to estimate the relative proportion of cell types in the mixed cell populations[47]. We used this method to explore relative fractions of the 22 leukocyte subtypes in peripheral blood of patients with MMD and healthy subjects (**Fig 4A**). As a result, we found that MMD patients exhibited significant increase of naïve B cells and naïve CD4 cells, as well as significant decrease of resting natural killer cells compared to healthy controls (**Fig 4B**). As is known, keeping adequate amount of circulating leukocytes is essential to the immune system. Disturbance of these three leukocyte subtypes and other cell types like EPCs and smooth muscle cells may together contribute to the vascular stenosis or occlusion in MMD. Nevertheless, the exact molecular mechanism whereby these disturbed cell types contribute to the progression of MMD warrants to be investigated in the future.

MMD is a unique cerebrovascular disease which can result in ischemic and/or hemorrhagic events in the brain, sharing many common clinical characteristics with other vascular diseases. Association of MMD with other vascular disorders including atherosclerotic disease and coronary heart disease were widely reported[22–29]. Therefore, it is of great interest to compare MMD with other vascular disorders. In this study, we for the first time compared MMD with other vascular disorders including IS, ATS, fHC, CAD, and MI from the perspective of peripheral blood transcriptome. We found that few DEGs and enriched biological processes in GO analysis were shared by MMD and other vascular disorders. According to GSEA analysis, genes up-regulated in IS were over-represented in up-regulated genes of MMD, while down-regulated genes of CAD and MI were enriched in down-regulated genes of MMD (**Fig 5**). These findings suggested that MMD was only partially analogous to IS, CAD and MI on the peripheral blood transcriptomic level, and this can be partially explained by the disease causes and clinical symptoms. IS refers to a cerebral hypoxia state due to insufficient blood following to the brain, eventually leading to brain dysfunction such as unconsciousness and impairments in vision or speaking. Given that cerebral ischemia is also a common clinical manifestation of MMD resulting from stenosis or occlusion at the terminal portion of ICA, it is very likely that MMD could display a certain similarity with IS on gene expression pattern in peripheral blood and this similarity is mainly related to those genes involved in ECM organization. CAD belongs to the group of cardiovascular disease, while MI is the most serious type of CAD. Clinical symptoms including chest pain, shortness of breath, abnormal heartbeat, and heart failure usually occur in patients with coronary heart disease. CAD was considered to be just a simple lipid accumulation disease in the past, while now growing studies have suggested that inflammation could play a vital role in all the stage of CAD development[68–71]. Inflammatory markers of CAD were therefore widely investigated, which was useful for estimating disease risk[72–76]. Hence, inflammation may partly underlie the similarity between MMD and CAD as well as MI regarding transcriptomic profiles in peripheral blood. In addition, cases of CAD in patients with MMD have been extensively reported[24–29].Therefore, it may hint that MMD is a systemic vasculopathy that both circulating inflammatory factors and the imbalance of cell populations in the blood are to blame for the progressive narrowing or occlusion of the involved arteries. Also, it is likely that MMD could lead to occlusion of coronary arteries through certain systemic etiologic factors in the blood. Despite sharing certain similarities with other vascular diseases, MMD exhibited a unique gene expression pattern perhaps due to the specific pathogenic mechanism and the very disease initial site. Specially, a fine vascular network forms at the base of the brain to compensate decreased blood supply in MMD patients, which does not exist in patients with other vascular disorders. In summary, our findings suggested that compared with other vascular disorders, gene dysregulation in peripheral blood of MMD was specific and mainly associated with ECM organization, immune and inflammatory responses.

Nevertheless, this is an exploratory study. It should be noted that there are some limitations in the present study. First, the relative small sample size in our RNA-Seq experiment may exert a certain influence on the robustness of our results, although we validated some of the DEGs involved in enriched biological pathways in independent samples with relatively larger sample size. Second, we did not consider the differences between MMD patients with different initial symptoms. Future studies comparing gene expression pattern in peripheral blood of MMD patients with different manifestations are necessary and important to elucidate the heterogeneity of MMD. In addition, public datasets used in the study contain gene expression profiles from different profiling platforms, although strict preprocessing and normalization of data have been made. Last, results of cell type deconvolution warrant experimental validation with effective markers which can accurately discriminate the functional state of immune cells in the future. Also, applications of disease-specific leucocyte subtypes derived from induced pluripotent stem cell and single-cell RNA-Seq technology could be a promising approach to study the potential roles of disrupted cell types in peripheral blood of MMD patients.

## Conclusion

In conclusion, the present study demonstrated that dysregulated genes in peripheral blood of MMD patients mainly played key roles in ECM organization, immune and inflammatory responses. This gene dysregulation pattern in peripheral blood was specific for MMD compared with other vascular disorders. Besides, proportion of naïve B cells, naïve CD4 cells and resting natural killer cells in peripheral blood showed a significant difference between MMD patients and healthy subjects, which may hint multiple cell types were involved in the development of MMD. Our study will help elucidate the unclear pathogenesis of MMD and clarify the complicated association between MMD and other vascular disorders.

## Supporting information

S1 Fig Validation of DEGs in independent male and female samples. **(A-B)** Validation of DEGs using qRT-PCR in independent male (MMD patients, n = 6; healthy controls, n = 15) and female (MMD patients, n = 11; healthy controls, n = 8) samples, respectively. Subjects with hypertension, hyperlipidemia, and diabetes mellitus were removed. Normalized fold expression of mRNA are shown. Expression values are first normalized to *B2M* (internal control) and then plotted relative to corresponding healthy controls that are set as 1 for each gene. Error bar represents mean ± SEM (n = 3 independent experiments). Unpaired student’s *t*-test is used to calculate significant differences (*p < 0.05, **p < 0.01). MMD = moyamoya disease; DEGs = differentially expressed genes.
(TIF)

S1 Table Datasets for vascular disorders. IS = ischemic stroke; ATS = atherosclerosis; fHC = familial hypercholesterolemia; CAD = coronary artery disease; MI = myocardial infarction.
(DOCX)

S2 Table Primers for qRT-PCR. (DOCX)

S3 Table Data of other clinical tests on MMD patients. MMD = moyamoya disease; TC = total cholesterol; TG = triglycerides; anti-M2 = anti-mitochondrial M2; anti-SSA = anti-Sjögren’s-syndrome-related antigen A; anti-PM-Scl = anti-polymyositis/systemic sclerosis; T3 = triiodothyronine; T4 = thyroxine; TSH = thyroid-stimulating hormone; FT3 = free triiodothyronine; FT4 = free thyroxine; FC = folic acid; VB_12_ = vitamin B_12_; ↔ = normal level; ↑ = high level; ↓ = low level;— = no data. Reference ranges: TC (2.33–6.20mmol/L); TG (0.45–1.81mmol/L); T3 (1.02–2.69nmol/L); T4 (55.50–161.30nmol/L); TSH (0.51–4.94μIU/mL); FT3 (2.80–6.30pmol/L); FT4 (11.50–22.70pmol/L); FC (3.89–26.80ng/mL); VB_12_ (197.00–771.00pg/mL).
(DOCX)

S4 Table Number of DEGs identified in each vascular disorder and overlap between each other. DEGs = differentially expressed genes; MMD = moyamoya disease; IS = ischemic stroke; ATS = atherosclerosis; fHC = familial hypercholesterolemia; CAD = coronary artery disease; MI = myocardial infarction.
(DOCX)

S1 File Summary of enriched GO terms for up-regulated and down-regulated genes in different vascular disorders. GO = Gene Ontology; DEGs = differentially expressed genes; MMD = moyamoya disease; IS = ischemic stroke; ATS = atherosclerosis; fHC = familial hypercholesterolemia; CAD = coronary artery disease; MI = myocardial infarction. GO terms with *p* < 0.05 are selected.
(XLSX)
