# Supplementary figures and images for: Gene dysregulation in peripheral blood of moyamoya disease and comparison with other vascular disorders
Source: PLoS One. 2019 Sep 18;14(9):e0221811. doi: 10.1371/journal.pone.0221811 (PMC6750579; doi:10.1371/journal.pone.0221811)

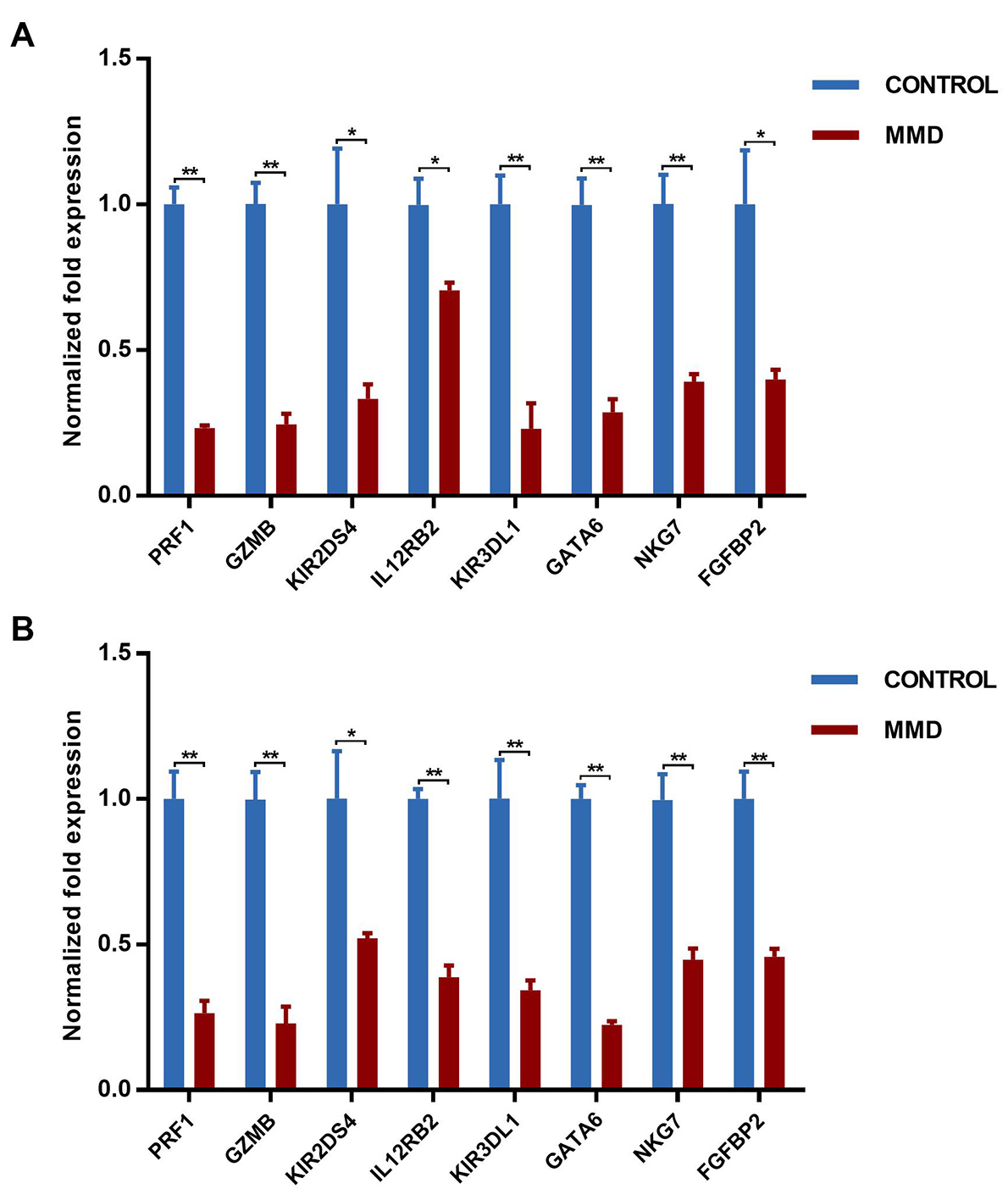

Supplement: S1 Fig — (A-B) Validation of DEGs using qRT-PCR in independent male (MMD patients, n = 6; healthy controls, n = 15) and female (MMD patients, n = 11; healthy controls, n = 8) samples, respectively. Subjects with hypertension, hyperlipidemia, and diabetes mellitus were removed. Normalized fold expression of mRNA are shown. Expression values are first normalized to B2M (internal control) and then plotted relative to corresponding healthy controls that are set as 1 for each gene. Error bar represents mean ± SEM (n = 3 independent experiments). Unpaired student’s t-test is used to calculate significant differences (*p < 0.05, **p < 0.01). MMD = moyamoya disease; DEGs = differentially expressed genes. (TIF) [file pone.0221811.s001.tif]
