# Supplementary material for: Gene dysregulation in peripheral blood of moyamoya disease and comparison with other vascular disorders
Source: PLoS One. 2019 Sep 18;14(9):e0221811. doi: 10.1371/journal.pone.0221811 (PMC6750579; doi:10.1371/journal.pone.0221811)
Supplement: S1 Table — IS = ischemic stroke; ATS = atherosclerosis; fHC = familial hypercholesterolemia; CAD = coronary artery disease; MI = myocardial infarction. (DOCX) [file pone.0221811.s002.docx]

**S1 Table. Public datasets for vascular disorders.**

| **Disease** | **GEO accession** | **Platform** | **No. of cases** | **No. of controls** |
| --- | --- | --- | --- | --- |
| IS | GSE16561 | GPL6883 | 39 | 24 |
| ATS | GSE20129 | GPL10558 | 8 | 8 |
| fHC | GSE13985 | GPL570 | 5 | 5 |
| CAD | GSE42148 | GPL13607 | 13 | 11 |
| MI | GSE60993 | GPL6884 | 6 | 7 |
|  | GSE61144 | GPL6106 | 7 | 10 |

IS = ischemic stroke; ATS = atherosclerosis; fHC = familial hypercholesterolemia; CAD = coronary artery disease; MI = myocardial infarction; GEO = gene expression omnibus.
