# Supplementary material for: Gene dysregulation in peripheral blood of moyamoya disease and comparison with other vascular disorders
Source: PLoS One. 2019 Sep 18;14(9):e0221811. doi: 10.1371/journal.pone.0221811 (PMC6750579; doi:10.1371/journal.pone.0221811)
Supplement: S2 Table — (DOCX) [file pone.0221811.s003.docx]

**S2 Table. Primers for qRT-PCR.**

| **Gene symbol** | **Forward (5’ to 3’)** | **Reverse (5’ to 3’)** |
| --- | --- | --- |
| FBN2 | ACCTCAACAGATGGCTCTCG | ACAGCAGCACTGCATTTTCG |
| KIF26B | CTGTGATGAGGACGACCACC | ACCTCCAGACATTCCCCCTT |
| PRF1 | ACTCACAGGCAGCCAACTTT | GCCCTCTTGAAGTCAGGGTG |
| GZMB | GAGCAAGGAGGAAACAACAGC | TGATCTCCCCTGCATCTGCC |
| KIR2DS4 | CGCAGCATCAACGGAACATT | TGACGGAAACAAGCAGTGGA |
| IL12RB2 | ACCTCCTGGACCACAGTCAT | CAGCAACCCTGCCTCACA |
| KIR3DL1 | CAGTGGTCATCATCCTCTTCATCC | TTCATCAGAGTCCTCGCTGTTGG |
| GATA6 | AGAAGCGCGTGCCTTCATC | TTTCTGCGCCATAAGGTGGT |
| NKG7 | GGACATCATATCAGGCTACATCC | GACAGGACCAGGAAGCTCAC |
| FGFBP2 | GGTGACAGGTGAAAGACCCC | CGTTGGATTGAAAGCGGCAT |
| B2M | AGATGAGTATGCCTGCCGTG | TCAAACCTCCATGATGCTGCT |
