# Supplementary material for: Gene dysregulation in peripheral blood of moyamoya disease and comparison with other vascular disorders
Source: PLoS One. 2019 Sep 18;14(9):e0221811. doi: 10.1371/journal.pone.0221811 (PMC6750579; doi:10.1371/journal.pone.0221811)
Supplement: S3 Table — MMD = moyamoya disease; TC = total cholesterol; TG = triglycerides; anti-M2 = anti-mitochondrial M2; anti-SSA = anti-Sjögren’s-syndrome-related antigen A; anti-PM-Scl = anti-polymyositis/systemic sclerosis; T3 = triiodothyronine; T4 = thyroxine; TSH = thyroid-stimulating hormone; FT3 = free triiodothyronine; FT4 = free thyroxine; FC = folic acid; VB12 = vitamin B12; ↔ = normal level; ↑ = high level; ↓ = low level;— = no data. Reference ranges: TC (2.33–6.20mmol/L); TG (0.45–1.81mmol/L); T3 (1.02–2.69nmol/L); T4 (55.50–161.30nmol/L); TSH (0.51–4.94μIU/mL); FT3 (2.80–6.30pmol/L); FT4 (11.50–22.70pmol/L); FC (3.89–26.80ng/mL); VB12 (197.00–771.00pg/mL). (DOCX) [file pone.0221811.s004.docx]

**S3 Table. Data of other clinical tests on MMD patients.**

| **Sample** | **TC** | **TG** | **Antinuclear antibody spectrum** | **Thyroid function** | **Thyroid autoantibodies** | **Anti-O & rheumatoid factors** | **Serum folic acid & vitamin B_12_** |
| --- | --- | --- | --- | --- | --- | --- | --- |
| MMD1 | 2.49 | 1.16 | ↔ | ↔ | ↔ | - | FC2.46↓ |
| MMD2 | 3.10 | 0.72 | anti-histone antibody(+) | FT_3_6.9↑ | - | - | FC1.85↓ |
| MMD3 | 3.94 | 1.18 | anti-PM-Scl antibody(++) | ↔ | ↔ | - | VB_12_＞2000.0↑ |
| MMD4 | 2.51 | 1.59 | ↔ | TSH5.34↑ | ↔ | - | ↔ |
| MMD5 | 4.60 | 1.66 | ↔ | - | - | - | VB_12_945.0↑ |
| MMD6 | 3.87 | 1.76 | ↔ | ↔ | ↔ | ↔ | FC2.56↓ |
| MMD7 | 4.67 | 1.37 | ↔ | ↔ | ↔ | - | ↔ |
| MMD8 | 3.61 | 1.31 | ↔ | ↔ | ↔ | - | FC1.89↓VB_12_1093.0↑ |
| MMD9 | 3.87 | 0.80 | ↔ | ↔ | - | - | FC2.57↓VB_12_1163.0↑ |
| MMD10 | 4.27 | 0.90 | ↔ | ↔ | ↔ | - | FC3.42↓ |
| MMD11 | 4.00 | 0.76 | ↔ | ↔ | ↔ | - | ↔ |
| MMD12 | 4.88 | 1.55 | ↔ | ↔ | ↔ | - | ↔ |
| MMD13 | 3.15 | 0.78 | anti-histone antibody(++) | ↔ | - | - | FC1.88↓ |
| MMD14 | 3.29 | 1.13 | ↔ | ↔ | ↔ | - | ↔ |
| MMD15 | - | - | ↔ | TSH6.59↑ | ↔ | - | - |
| MMD16 | 2.86 | 2.82↑ | ↔ | ↔ | ↔ | ↔ | ↔ |
| MMD17 | 4.83 | 1.42 | ↔ | ↔ | ↔ | - | ↔ |
| MMD18 | 5.60 | 1.66 | ↔ | ↔ | - | - | - |
| MMD19 | 3.13 | 1.76 | ↔ | - | - | - | ↔ |
| MMD20 | 4.21 | 1.56 | anti-M2 antibody(++) | ↔ | - | - | - |
| MMD21 | - | - | ↔ | ↔ | - | - | - |
| MMD22 | 4.86 | 1.69 | ↔ | ↔ | - | - | FC2.37↓ |
| MMD23 | 4.42 | 1.00 | ↔ | FT_4_10.7↓TSH5.19↑ | ↔ | - | ↔ |
| MMD24 | 2.65 | 1.00 | ↔ | ↔ | ↔ | - | FC1.47↓ |
| MMD25 | 1.85↓ | 0.55 | anti-SSA antibody(+++) | ↔ | ↔ | - | VB_12_1915.0↑ |
| MMD26 | 3.37 | 1.42 | - | - | - | - | ↔ |
| MMD27 | 4.50 | 0.65 | - | ↔ | ↔ | - | VB_12_1836.0↑ |
| MMD28 | 4.20 | 0.72 | anti-SSA antibody(+) | - | - | - | - |
| MMD29 | 3.89 | 1.24 | ↔ | ↔ | - | - | ↔ |
| MMD30 | 2.58 | 1.28 | ↔ | ↔ | - | - | ↔ |
| MMD31 | 5.16 | 1.11 | ↔ | FT_3_6.7↑ | - | - | ↔ |
| MMD32 | 3.69 | 1.74 | ↔ | ↔ | - | - | - |
| MMD33 | 3.01 | 0.83 | ↔ | ↔ | ↔ | - | - |
| MMD34 | 4.37 | 1.39 | ↔ | ↔ | - | - | FC3.58↓ |
| MMD35 | 2.42 | 0.58 | ↔ | - | - | - | FC3.41↓ |

MMD = moyamoya disease; TC = total cholesterol; TG = triglycerides; anti-M2 = anti-mitochondrial M2; anti-SSA = anti-Sjögren’s-syndrome-related antigen A; anti-PM-Scl = anti-polymyositis/systemic sclerosis; T3 = triiodothyronine; T4 = thyroxine; TSH = thyroid-stimulating hormone; FT3 = free triiodothyronine; FT4 = free thyroxine; FC = folic acid; VB_12_ = vitamin B_12_; ↔ = normal level; ↑ = high level; ↓ = low level; - = no data.

Reference ranges: TC (2.33-6.20mmol/L); TG (0.45-1.81mmol/L); T3 (1.02-2.69nmol/L); T4 (55.50-161.30nmol/L); TSH (0.51-4.94μIU/mL); FT3 (2.80-6.30pmol/L); FT4 (11.50-22.70pmol/L); FC (3.89-26.80ng/mL); VB_12_ (197.00-771.00pg/mL).
