# Supplementary material for: Gene dysregulation in peripheral blood of moyamoya disease and comparison with other vascular disorders
Source: PLoS One. 2019 Sep 18;14(9):e0221811. doi: 10.1371/journal.pone.0221811 (PMC6750579; doi:10.1371/journal.pone.0221811)
Supplement: S4 Table — DEGs = differentially expressed genes; MMD = moyamoya disease; IS = ischemic stroke; ATS = atherosclerosis; fHC = familial hypercholesterolemia; CAD = coronary artery disease; MI = myocardial infarction. (DOCX) [file pone.0221811.s005.docx]

**S4 Table. Number of DEGs identified in each vascular disorder and overlap between each other.**

| **Disease** | **MMD** | **IS** | **ATS** | **fHC** | **CAD** | **MI** |
| --- | --- | --- | --- | --- | --- | --- |
| **MMD** | 533 | 18 | 0 | 15 | 27 | 29 |
| **IS** | - | 341 | 8 | 32 | 36 | 159 |
| **ATS** | - | - | 43 | 4 | 4 | 11 |
| **fHC** | - | - | - | 389 | 31 | 45 |
| **CAD** | - | - | - | - | 786 | 75 |
| **MI** | - | - | - | - | - | 616 |

DEGs = differentially expressed genes; MMD = moyamoya disease; IS = ischemic stroke; ATS = atherosclerosis; fHC = familial hypercholesterolemia; CAD = coronary artery disease; MI = myocardial infarction.
